# Respiratory Syncytial Virus Resurgence in Italy: The Need to Protect All Neonates and Young Infants

**DOI:** 10.3390/ijerph19010380

**Published:** 2021-12-30

**Authors:** Elena Bozzola

**Affiliations:** Pediatric Unit, Bambino Gesù Children’s Hospital, IRCCS, 00100 Rome, Italy; elena.bozzola@opbg.net; Tel.: +39-064-938-2744

Respiratory syncytial virus (RSV) is the most prevalent cause of viral respiratory infections in children up to the age of 2 years and causes a wide range of clinical manifestations [1]. It circulates seasonally with variable epidemiology, depending on geographic area and climate. In temperate regions of the Northern Hemisphere, such as Italy, the virus diffusion generally occurs in late fall and early spring, with a peak incidence in January/February. Infants born prematurely or close to the RSV season and/or suffering from bronchopulmonary dysplasia or congenital heart disease have the highest risk of developing severe RSV-related acute LRTI. Nevertheless, all infants are at risk of RSV infection, as epidemiological studies reveal that more than 60% of all children are infected by RSV within the first year of age, and approximately all children are infected within the second years of age [1,2,3].

A broad spectrum of disease presentation is associated with RSV infection, such as bronchiolitis and pneumoniae, ranging from mild to severe symptoms, which may require hospitalization for intensive care unit admission or for oxygen supplementation in pediatric wards. Notably, a high rate of pediatric visits may be required both in the acute phase and in the follow-up period due to the increased risk of long-term morbidities. Evidence suggests that children who experienced RSV infection early in life are at risk of developing wheezing, hyperactive airways, bronchospasm, and asthma later in life. Overall, these factors contribute to substantial health care use and economic burden of RSV disease long-term complications, caregiver stress, and loss of work productivity [4,5]. To date, no effective and specific treatment is available to cure RSV, as therapeutic measures are still undergoing preliminary research. There is only a single antiviral drug currently licensed, but due to questionable efficacy, side effects, and cost, it is recommended only for high-risk patients. Therefore, despite the large medical and economic burden, treatment of RSV disease remains largely symptomatic, and supportive care consists of supplementing oxygen therapy, nutrition, and fluids. Aside from therapeutic measures, preventive strategies are also still in developing stages, except for a limited group of high-risk children.

Currently, the only drug available to prevent RSV infections is a humanized monoclonal antibody (mAb), palivizumab, directed against a neutralizing epitope of the RSV F protein. The administration of palivizumab is strictly regulated by directives from the Italian Medicine Agency (AIFA), which are based on the evidence from the literature, the recommendations formulated by scientific societies, and the characteristics of the product as indicated by the industry.

In Italy, the use of palivizumab is restricted to infants born at <29 weeks of gestation and less than one year of age at the beginning of the RSV season or infants with specified several risk factors. Additionally, the high cost of palivizumab and the short half-life, approximately 20 days, which requires monthly administrations during the epidemic period, represent a further limit to an extensive use of the product. On the other hand, there is a need for specific products to provide prolonged and effective protection throughout the RSV epidemic season to all neonates and infants regardless of gestational age at birth [4].

The need is even stronger in recent months, since the start of the COVID-19 pandemic. At the beginning of the emergency, public health measures revealed that the control of SARS-CoV-2 modified the epidemiology of other respiratory viruses including RSV. Consequently, in Italy, as well as in other countries, a strong reduction in all viral respiratory infections was observed in the last season (2020–2021), compared with the previous seasons [5,6,7].

Nevertheless, after a relative absence during the past season, a large resurgence of RSV detections occurred in recent months in Italy. In fact, once the severely restrictive public health measures were reduced, RSV found a large pool of susceptible babies and children to infect, leading to sudden surges and raising concern for the Italian health system to face this epidemic. This event further underlines the need for an effective and specific prevention strategy to prevent RSV in all neonates and young infants.

To date, there is no approved specific vaccine to prevent RSV in all neonates and infants, even if its development was among the priorities of the World Health Organization [8]. Young infants represent the main target for vaccination as the peak of severe RSV disease is in the first 3 months of life. Nevertheless, there are concerns that anti-RS responses may be non-effective, especially in the youngest infants compromised by maternal antibodies, which may interfere with vaccine immunogenicity. Moreover, the immaturity of the immune system of the infants in the first months of life may reduce vaccine efficacy [9]. Therefore, pediatric vaccines may be offered to children during infancy, but they would not be able to protect them in the first months of life, in which the risk of RSV hospitalization is higher. This event may represent a limitation to the product for those infants born during RSV season, as they may be exposed to the risk of infection and complications.

Another approach may be based on maternal immunization, with the goal of protecting neonates from RSV through passive transfer of maternal antibodies. Through pregnant women immunization and subsequent transplacental transfer of maternal antibodies, neonates may receive protection during the first months of life. Nevertheless, the maternal immunity induced by vaccines may not translate into sufficient protection of the baby, mainly in case of premature birth because active transplacental antibody transfer begins at approximately 28 to 30 weeks of gestation. Additionally, the maternal vaccination strategy may be considered only for children born coincidentally with the RSV epidemic season but not for those born in the previous months for the short duration of maternal antibodies [9].

Another strategy considers passive immunoprophylaxis with an extended serum half-life mAb lasting up to 5 months to cover the entire RSV season by a single intramuscular injection. This therapeutical approach may consider the mAb administration to the neonates before hospital discharge if born during the epidemic season or during a follow-up appointment outside the season. Beyond having a long-lasting and effective neutralizing activity, passive rather than active vaccination may also be a good option in the case of mAb treatment having a vaccine-comparable pricing, to protect all neonates and infants from RSV infection [4,9].

## Data Availability

Bambino Gesù Children Hospital, Bozzola’s repository.

## Abbreviations

RSV	Respiratory syncytial virus
mAb	Monoclonal antibody
